# Taxonomic Description and Genome Sequence of *Christensenella intestinihominis* sp. nov., a Novel Cholesterol-Lowering Bacterium Isolated From Human Gut

**DOI:** 10.3389/fmicb.2021.632361

**Published:** 2021-02-22

**Authors:** Yuanqiang Zou, Wenbin Xue, Xiaoqian Lin, Tongyuan Hu, Shao-Wei Liu, Cheng-Hang Sun, Guangwen Luo, Mei Lv, Ying Dai, Karsten Kristiansen, Liang Xiao

**Affiliations:** ^1^BGI-Shenzhen, Shenzhen, China; ^2^Laboratory of Genomics and Molecular Biomedicine, Department of Biology, University of Copenhagen, Copenhagen, Denmark; ^3^Shenzhen Engineering Laboratory of Detection and Intervention of Human Intestinal Microbiome, BGI-Shenzhen, Shenzhen, China; ^4^Qingdao-Europe Advanced Institute for Life Sciences, BGI-Shenzhen, Qingdao, China; ^5^School of Bioscience and Biotechnology, South China University of Technology, Guangzhou, China; ^6^Institute of Medicinal Biotechnology, Chinese Academy of Medical Sciences and Peking Union Medical College, Beijing, China; ^7^BGI College and Henan Institute of Medical and Pharmaceutical Sciences, Zhengzhou University, Zhengzhou, China

**Keywords:** *Christensenella*, *Christensenella intestinihominis* sp. nov., taxonomy, genome sequencing, phylogenetic analysis, cholesterol-lowering

## Abstract

A Gram-staining-negative, non-spore-forming, short, straight rod, non-motile, and obligate anaerobic bacterial strain, AF73-05CM02^T^, was isolated from a fecal sample of a 30 years old healthy male living in Shenzhen, China. Colonies were approximately 0.2 mm in diameter, beige, and circular after 4 days of incubation on PYG agar under anaerobic conditions at 37°C. Strain AF73-05CM02^T^ grew in a temperature range between 30 and 42°C and a pH range from 6.0 to 8.5, with optimum growth at 37–42°C and pH 7.0. 16S rRNA gene sequence analysis demonstrated that strain AF73-05CM02^T^ belongs to the genus *Christensenella* and showed the highest level of sequence similarity (98.68%) with *Christensenella minuta* DSM 22607^T^. The predominant fatty acids of strain AF73-05CM02^T^ were C_10__:__0_ (7.5%), iso-C_11__:__0_ (5.6%), C_12__:__0_ (7.2%), C_14__:__0_ (46.6%), iso-C_15__:__0_ (7.4%), C_16__:__0_ (9.7%), and C_18__:__1_ ω9*c* (6.9%). Acetic acid, formic acid, butyric acid, and lactic acid were the end products of glucose fermentation. The strain was negative for catalase, indole production, and hydrolysis of gelatin. Genomic relatedness analyses based on average nucleotide identity (ANI) indicated that strain AF73-05CM02^T^ significantly differed from other species of the genus *Christensenella*, showing ANI values less than 82.89% with the phylogenetically closest species. The G + C content of the genomic DNA was 52.07 mol% from the genome sequence, which differs from that of *Christensenella minuta*. Several physiological, biochemical, and genotypic properties differentiated the novel bacterial strain from the related species, indicating that the strain represents a new species of the genus *Christensenella* for which the name *Christensenella intestinihominis* sp. nov. is proposed, with strain AF73-05CM02^T^ ( = CGMCC 1.5207^T^ = DSM 103477^T^ ) being the type strain. The following study explored the cholesterol-lowering function of strains AF73-05CM02^T^ and *Christensenella minuta* DSM 22067^T^ and revealed that the two strains exhibit the capacity for removing cholesterol with efficiency rates of 36.6 and 54.3% and produce exopolysaccharide of 234 and 271 mg/L, respectively.

## Introduction

The human gut is colonized by a large and complex community of microorganisms ranging from 10^13^ to 10^14^ microbial cells (Ventura, 2009; Ghosh, 2013), which is equivalent to 10 times the number of human cells (Bäckhed et al., 2005; Jeffery et al., 2016). Colonization of the intestinal tract begins shortly after birth, and the gut microbiota develops over the first few years (Palmer et al., 2007). The composition of the microbiota is affected by many factors, including the genetic background of the host (Khachatryan et al., 2008; Benson et al., 2010; Fan et al., 2020), the immune status (Hooper et al., 2012), and living condition and daily diet (Turnbaugh et al., 2009; Fujimura et al., 2010). The two bacterial phyla, Firmicutes and Bacteroidetes make up about 90% of the gut microbiota (Turnbaugh et al., 2006, 2008; Tremaroli and Backhed, 2012). *Christensenella minuta* YIT 12065^T^, the type species of the genus *Christensenella* within the family Christensenellaceae, isolated from human feces, was first described in 2012 (Morotomi et al., 2012). Phylogenetically, the strain formed a novel family-level lineage within the order Clostridiales with 86.9–86.1% 16S rRNA gene sequence similarity with the closest relatives. *C. minuta* YIT 12065^T^ was identified as a Gram-negative, non-motile, non-spore-forming, short, straight rod with tapered ends, which grew anaerobically. The major fatty acids are iso-C_15__:__0_, C_14__:__0_, and C_16__:__0_. LL-Diaminopimelic acid is present in the cell wall. The draft genome of *C. minuta* YIT 12065^T^ has been reported previously (Rosa et al., 2017; Coil et al., 2020). *C. minuta* has been identified as a beneficial bacteria protecting against obesity (Goodrich et al., 2014).

Cholesterol is an important basic substance for the human body. However, an elevated level of blood cholesterol increases the risk of cardiovascular diseases (CVDs) (Tok and Aslim, 2010; Tsai et al., 2014), which remain a leading cause of deaths worldwide (Ishimwe et al., 2015). In recent years, probiotics have been developed as a non-drug therapy to reduce blood lipids and cholesterol levels and the risk of CVDs (Pan et al., 2010; Tsai et al., 2014). Several mechanisms have been proposed for explaining the cholesterol-lowering effect of different probiotics, including deconjugation of bile by bile salt hydrolase activity (Lye et al., 2010a), assimilation and conversion of cholesterol by probiotics (Gilliland et al., 1985; Lye et al., 2010b), and modulation of cholesterol absorption in the intestines of the host (Huang and Zheng, 2010; Yoon et al., 2013).

In the present study, we focus on a polyphasic taxonomic approach for a novel strain, *C. intestinihominis* sp. nov. AF73-05CM02^T^, along with the whole genome sequencing and annotation data, and further investigated its cholesterol-lowering property.

## Materials and Methods

### Strain Isolation

The fresh fecal sample was collected from a healthy adult living in Shenzhen, China, and brought back to the laboratory and then used for isolation of bacteria. For cultivation, approximately 1 g fresh fecal material was transferred into an anaerobic box (Bactron Anaerobic Chamber, Bactron IV-2, Shel Lab, United States) with a gas phase of N_2_/H_2_/CO_2_ (90:5:5, v/v) and dispersed in 0.1 M PBS (pH 7.0). This suspension containing bacteria was mixed thoroughly and serially diluted and spread onto peptone-yeast extract-glucose (PYG) plates as described previously (Zou et al., 2019). The plates were incubated at 37°C for 1 week under anaerobic condition. Single colonies were picked and purified by inoculation and subculturing on the same medium. In this study, one of these strains, designated AF73-05CM02^T^, was maintained as a glycerol suspension (20%, w/v) at −80°C.

### 16S rRNA Gene Sequencing and Phylogenetic Analysis

The genomic DNA of strain AF73-05CM02^T^ was prepared from cells harvested from PYG broth using the phenol: chloroform method (Cheng and Jiang, 2006). The 16S rRNA gene was amplified using the universal bacterial primers 27F–1492R (5′-AGAGTTTGATCATGGCTCAG-3′ and 5′-TAGGGTTACCTTGTTACGACTT-3′) and purified as described by Zou et al. (2013). Sequencing was performed by BGI-Shenzhen (Shenzhen, China). The resulting sequence was compared with sequences of type strains retrieved from the EzBioCloud server (Yoon et al., 2017)^1^ using BLAST. Phylogenetic analysis was performed using software package MEGA X (Kumar et al., 2018) after multiple alignment of sequences data by using the CLUSTALW program (Thompson et al., 1994). Evolutionary phylogenetic trees were constructed using the neighbor-joining method (Saitou and Nei, 1987), the maximum-likelihood (Felsenstein, 1981) method, and the minimum-evolution method (Rzhetsky and Nei, 1993), and bootstrap values were calculated based on 1,000 replications.

### Genome Sequencing, GC Content, and Genome Comparison

For genome comparison of the novel strain and the closely related species, we conducted genome sequencing and assembly of strain AF73-05CM02^T^. Draft genome sequencing was carried out using a paired-end sequencing strategy with Ion Proton Technology (Life Technologies) at BGI-Shenzhen (Shenzhen, China). The paired-end library had a mean insert size of 500 bp. Reads were assembled using the SOAPdenovo 2 package (Luo et al., 2012). The genomic DNA base content (mol% G + C) was directly calculated from the draft genome data. To determine the DNA relatedness between strain AF73-05CM02^T^ and the most closely related species, *C. minuta* DSM 22607^T^ and *Catabacter hongkongensis* HKU16^T^ (Lau et al., 2007, 2015), we calculated the average nucleotide identity values (Damodharan et al., 2015), which is considered to correspond to DNA–DNA hybridization (Goris et al., 2007; Tindall et al., 2010) as described by Kim et al. (2014), following the BLAST-based ANI calculation using the EzGenome web service. ANI values of 95–96% corresponding to 70% DDH have been proposed as a threshold value for species delineation in bacterial taxonomy (Kim et al., 2014). The digital DNA–DNA hybridization (dDDH) values were calculated using the Genome-to-Genome Distance Calculator (GGDC) web tool^2^ (Auch et al., 2010). The obtained draft genome sequences were annotated using the Rapid Annotation Subsystem Technology (RAST) server (Kanehisa et al., 2016) and KEGG (Aziz et al., 2008) and COG databases (Galperin et al., 2015). A visual genomic comparison across strain AF73-05CM02^T^ and most closely related species was generated with the CGView server (Grant and Stothard, 2008)^3^. Analysis of genomic collinearity among strain AF73-05CM02^T^ and DSM 22607^T^ was conducted by the MCScanX software.

### Morphological Characteristics

Morphological characteristics were investigated with strain AF73-05CM02^T^ incubated in PYG medium at 37°C. Morphological observations were performed using a phase contrast microscopy (Olympus BX51, Japan). Gram staining, analysis of spore formation, and presence of flagella were performed by staining using the Gram stain kit (Solarbio), the spore stain kit (Solarbio), and the flagella stain kit (Solarbio) according to the manufacturer’s instructions. Cell motility was examined using semisolid PYG (0.4% agar) (Tittsler and Sandholzer, 1936). Colony morphology was observed for cultures grown on PYG agar for 4 days at 37°C. Growth at 4, 10, 20, 25, 30, 35, 37, 45, and 50°C was tested on PYG medium to determine the optimal temperature and temperature range for growth. The pH range for growth was evaluated at pH 3.0–10.0 (at an interval of 0.5 pH units) by adjusting the pH using the appropriate buffers as described by Sorokin (2005). Tolerance to NaCl was determined in PYG broth containing different concentrations of NaCl (0–6%, in increments of 1.0%). Bile tolerance was measured at different bile salt concentrations (0–5%, in increments of 1.0%) in the PYG broth containing all of the ingredients. All the growth tests of incubation under anaerobic conditions for 2 weeks was determined by measuring the OD_600_.

### Physiological and Biochemical Characteristics

Physiological and biochemical analyses comparing strain AF73-05CM02^T^ and the closely related species, *C. minuta* DSM 22607^T^, including measurements of enzyme activities, hydrolytic activities, utilization of various substrates as sole carbon sources, and acid production from different carbohydrates were carried out using the API ZYM, API 20A, and API 50CHL systems (bioMérieux, Marcy-l’Etoile, France). Sample preparation and test were performed following the manufacturer’s instructions with incubation at 37°C in an anaerobic chamber. For the API 50CHL test, CHL broth was supplied with 0.05% cysteine hydrochloride for cell suspension and incubation. Catalase activity was assessed in the presence of a 3% H_2_O_2_ solution using cells collected from colonies incubated on PYG agar at 37°C for 5 days (Smibert and Krieg, 1994). The strain and reference type strain were tested under the same laboratory conditions.

### Chemotaxonomical Characteristics

Chemotaxonomic characteristics of strain AF73-05CM02^T^ and the reference strain were performed by analyzing cellular fatty acids, cell wall composition, polar lipids, and quinones. Strains were cultured on PYG plates at 37°C for 2 days under anaerobic conditions, and fatty acid methyl esters (FAMEs) were prepared from lyophilized cells grown in the PYG medium by extraction and methylation as described previously (Chen and Dong, 2004). FAMEs were analyzed by an Agilent HP6890 gas chromatograph and identified using MIDI microbial identification system and performed by CGMCC (China General Microbiological Culture Collection Center, Beijing, China). The diagnostic isomer of diaminopimelic acid in whole-cell hydrolysates was identified by TLC as described by Zou et al. (2013). The polar lipids and quinones of strain AF73-05CM02^T^ and *C. minuta* DSM 22607^T^ were extracted from lyophilized bacterial cells and analyzed using two-dimensional TLC and HPLC coupled with a single quadrupole mass spectrometer (LCMS-2020, Shimadzu) as described (Liu et al., 2020).

### Susceptibility Tests and Hemolytic Activity

Susceptibility to antibiotics of strain AF73-05CM02^T^ was analyzed by the disk diffusion method according to Duran et al. (2012). Antibiotic disks (HANG WEI^TM^, China) were placed on PYG agar plates inoculated with prepared suspensions of the test organisms. The diameter of each zone was measured in millimeters after being incubated at 37°C for 5 days. The following antibiotic disks were tested: penicillin (10 μg), ampicillin (10 μg), carbenicillin (100 μg), vancomycin (30 μg), oxacillin (1 μg), piperacillin (100 μg), polymyxin B (300 IU), compound sulfamethoxazole (25 μg), furazolidone (300 μg), chloramphenicol (30 μg), and clindamycin (2 μg). Hemolytic activity was determined in sheep blood agar plates (Guangdong Huankai Microbial Sci. and Tech. Co., Ltd.). The plates were incubated under anaerobic conditions for 5 days at 37°C and checked for hemolysis (Pineiro and Stanton, 2007).

### Metabolic End Product Analysis

Identification of metabolic end products of glucose fermentation, including short-chain fatty acids (SCFAs) and organic acids, was performed using gas chromatograph (GC-7890B, Agilent) equipped with capillary columns and detected using a flame ionization detector (FID). The capillary column was packed with Agilent 19091N-133HP-INNOWax Porapak HP-INNOWax (30 m × 0.25 mm × 0.25 μm) for SCFA detection and Agilent 122-5532G DB-5ms (40 m × 0.25 mm × 0.25 μm) for other organic acids. The metabolic end products of strain AF73-05CM02^T^ were compared with the closely related species of the genus *Christensenella.*

### Property of Exopolysaccharide (EPS) Production

The functional properties of strains AF73-05CM02^T^ and *C. minuta* DSM 22607^T^ were determined by investigating the production of EPS. The EPS was isolated from the fermentation solution of the two strains using the method described previously (Mercan et al., 2015). In short, strains were inoculated in PYG broth at 37°C for 3 days, and the cultures were boiled at 100°C for 15 min. The bacterial supernatant was collected after centrifugation at 10,000*g* for 30 min at 4°C and treated with 80% trichloroacetic acid solution and stirred overnight for precipitating protein. The sample was centrifuged at 10,000*g* for 30 min at 4°C. The pH of the supernatant was adjusted to 7.0 with 2 M NaOH. A double volume of chilled ethanol was added to the supernatant, and EPS was precipitated overnight. The precipitated EPS was resuspended in distilled water with gentle heating. EPS was dialyzed using a 3,000 Da dialysis membrane for 24 h at 4°C and washed twice by distilled water. Total EPS production levels were determined using the phenol–sulfuric acid method with glucose as a standard (50–500 mg/L) (Dubois et al., 1956).

### Determination of Cholesterol-Lowering Activity

The capability of strain AF73-05CM02^T^ and the closely related reference strain *C. minuta* DSM 22607^T^ to lower cholesterol was determined according to a modified method of Damodharan et al. (2015). PYG-CHO broth was prepared with addition of 0.1% (w/v) bile, 0.2% (w/v) sodium thioglycollate, and cholesterol dissolved in ethanol to a final concentration of approximately 100 μg/ml. The PYG-CHO medium was inoculated with exponentially growing bacteria and incubated anaerobically at 37°C for 4 days. After incubation, cells were harvested by centrifugation at 10,000×*g* at 4°C for 10 min. The concentration of cholesterol in the supernatant was measured using the *o*-phthalaldehyde method as described by Rudel and Morris (1973).

Cholesterol-lowering activity from PYG-CHO of each strain broth was calculated in terms of percentage of cholesterol lowering as follows:

A=(B-C)/B*100%

where A = % of cholesterol lowering, B = the concentration of cholesterol in the PYG-CHO, and C = the concentration of cholesterol in the supernatant after being inoculated with bacteria for 4 days.

## Results and Discussion

### Strain Isolation

In the course of our ongoing investigation of the composition and diversity of the human gut microbiota using culture-dependent methods, we conducted a culturomics study using a fecal sample collected from a healthy adult using a nutrient-rich medium. Among the pure cultures grown on agar, a novel *Christensenella*-like strain, designated AF73-05CM02^T^, was selected for determination of its taxonomic position by using a polyphasic approach. The reference strain of the genus *Christensenella*, *C. minuta* DSM 22607^T^, procured from the Deutsche Sammlung von Mikroorganismen und Zellkulturen (DSMZ), Braunschweig, Germany, was used as reference strain for phenotypic characterization, genomic comparison, and analyses of cell fatty acids.

### Phylogeny Based on 16S rRNA Gene Sequences

**FIGURE 1:**
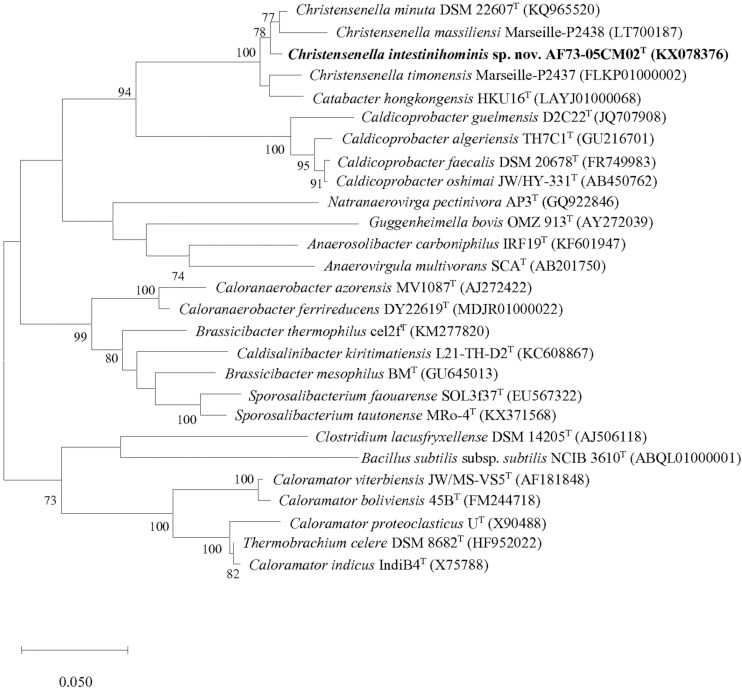
Maximum-likelihood phylogenetic tree based on 16S rRNA gene sequences showing the phylogenetic relationships of strain AF73-05CM02^T^ and the representatives of related taxa. *Bacillus subtilis* ssp. *subtilis* NCIB 3610^T^ (ABQL01000001) was used as an outgroup. Bootstrap values based on 1,000 replications higher than 70% are shown at the branching points. Bar, substitutions per nucleotide position.

We obtained the 16S rRNA gene sequence of strain AF73-05CM02^T^ (1,366 bp). The closest relatives of the strain were *C. minuta* DSM 22607^T^, *Catabacter hongkongensis* HKU16^T^ (Lau et al., 2007), “*Christensenella massiliensis*” Marseille-P2438 (Ndongo et al., 2016b), and “*Christensenella timonensis*” Marseille-P2437 (Ndongo et al., 2016a) with similarity values of 98.68, 97.22, 96.93, and 96.78%, respectively (Table 1). Phylogenetic analysis based on the maximum-likelihood algorithm confirmed the clustering of strain AF73-05CM02^T^ within the genus *Christensenella* and simultaneously formed a branch closest to *C. minuta* DSM 22607^T^ (Figure 1). The relationship between strain AF73-05CM02^T^ and the closest relatives was also found in a reconstructed tree using the neighbor-joining and maximum-likelihood algorithms (Supplementary Figures S1, S2). Strain AF73-05CM02^T^ shared a common branch with the closest relatives, *C. minuta* DSM 22607^T^, in all phylogenetic trees, demonstrating its evolutionary position within the genus *Christensenella*.

**TABLE 1:** Levels of 16S rRNA gene sequence similarity and ANI values (in percentages) based on BLAST for strain AF73-05CM02^T^ and the phylogenetically related species.

Strain	Accession no.	1	2*	3*	4*	5*
**16S rRNA gene sequence similarity (%)**						
AF73-05CM02^T^	KX078376	100				
*C. minuta* DSM 22607^T^	AB490809	98.68	100			
*Catabacter hongkongensis* HKU16^T^	AB671763	97.22	96.69	100		
“*C. massiliensis*” Marseille-P2438	LT161898	96.93	97.51	95.99	100	
“*C. timonensis*” Marseille-P2437	LT223568	96.78	97.38	96.79	95.40	100
ANI values (%)						
AF73-05CM02^T^	MAIQ00000000	100				
*C. minuta* DSM 22607^T^	NZ_CP029256	83.31	100			
*Catabacter hongkongensis* HKU16^T^	LAYJ00000000	73.84	75.39	100		
“*C. massiliensis*” Marseille-P2438	LT700187	78.00	78.01	73.28	100	
“*C. timonensis*” Marseille-P2437	FLKP00000000	74.06	74.56	74.53	73.59	100
**dDDH values (%)**						
AF73-05CM02^T^	MAIQ00000000	100				
*C. minuta* DSM 22607^T^	NZ_CP029256	26.80	100			
*Catabacter hongkongensis* HKU16^T^	LAYJ00000000	20.20	23.10	100		
“*C. massiliensis*” Marseille-P2438	LT700187	21.50	22.00	21.00	100	
“*C. timonensis*” Marseille-P2437	FLKP00000000	21.00	23.00	19.90	23.00	100

Taxa: 1, AF73-05CM02^T^; 2, C. minuta DSM 22607^T^; 3, Catabacter hongkongensis HKU16^T^; 4, “C. massiliensis” Marseille-P2438; 5, “C. timonensis” Marseille-P2437. *Data from NCBI and EzBioCloud.

### Genome Properties

**FIGURE 2:**
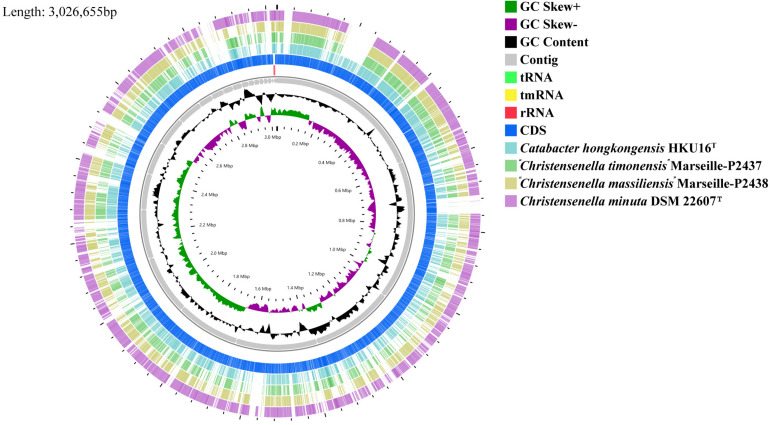
Graphical circular map of the genome from strain *Christensenella intestinihominis* sp. nov. AF73-05CM02^T^, *Christensenella minuta* DSM 22607^T^, *Catabacter hongkongensis* HKU16^T^, “*Christensenella massiliensis*” Marseille-P2438^T^, and “*Christensenella timonensis*” Marseille-P2437^T^ using the CGView server using default parameters. From inner to outer: Ring 1 and Ring 2, G + C positive skew (green) and G + C negative skew (purple), respectively; Ring 3, GC% content; Rings 4–8, Contig, rRNA, tmRNA, rRNA, and CDS from AF73-05CM02^T^, respectively; Ring 9, *Catabacter hongkongensis* HKU16^T^; Ring 10, “*Christensenella timonensis*” Marseille-P2437^T^; Ring 11, “*Christensenella massiliensis*” Marseille-P2438^T^; Ring 12, *Christensenella minuta* DSM 22607^T^.

The chromosome of strain AF73-05CM02^T^ was assembled from 3,145,728 reads resulting in a total length of 3,026,655 bp and comprising 29 scaffolds including 36 contigs. The G + C content of DNA for strain AF73-05CM02^T^ is 52.07 mol% as calculated from the whole-genome sequence. A circular map of strain AF73-05CM02^T^ in comparison to related species is shown in Figure 2. The general features of strain AF73-05CM02^T^ and the related species are summarized in Table 2.

**TABLE 2:** Genome features of *C. intestinihominis* AF73-05CM02^T^ and comparison with closely related species.

Features	1	2	3	4	5
Genome Size (bp)	3,026,655	2,969,292	3,151,949	2,560,186	2,650,850
G + C content (mol%)	52.07	51.4	48.5	50.4	51.7
DNA scaffolds	29	1	38	1	2
N50 length	294,532	2,969,292	166,940	2,560,186	2,314,156
Gene total number	2,642	2,875	2,986	2,515	2,483
rRNAs (5S, 16S, and 23S)	4	6	3	8	9
tRNAs	47	49	47	51	51
ncRNA	4	8	4	4	4
Genes assigned to COGs	2,176	ND	ND	ND	ND

*Taxa: 1, AF73-05CM02^T^; 2, C. minuta DSM 22607^T^; 3, Catabacter hongkongensis HKU16^T^; 4, “C. massiliensis” Marseille-P2438; 5, “C. timonensis” Marseille-P2437. ND, no data available.*

**FIGURE 3:**
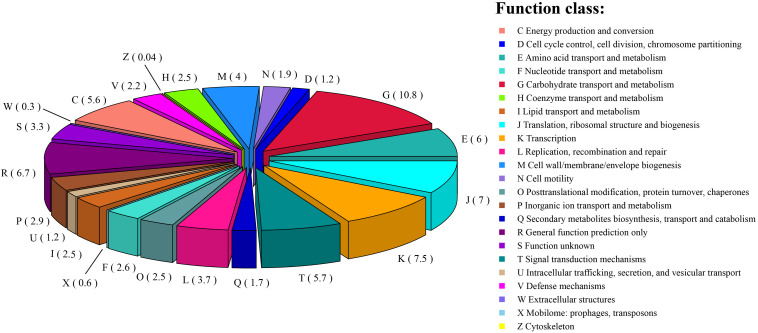
The distribution of the genes associated with the COG functional categories in strain AF73-05CM02^T^. The number of genes is shown in parentheses.

Among the 2,642 annotated genes in the *C. intestinihominis* AF73-05CM02^T^ genome, 2,176 genes with specific functions were assigned to COGs. The distribution of genes into COG functional classification is presented in Figure 3 and Supplementary Table S1, revealing that E (amino acid transport and metabolism), G (carbohydrate transport and metabolism), M (cell wall/membrane/envelope biogenesis), C (energy production and conversion), R (general function prediction only), T (signal transduction mechanisms), K (transcription), and J (translation, ribosomal structure, and biogenesis) were abundant categories. By analysis of the individual predicted coding sequences of strain AF73-05CM02^T^ using RAST annotation, we found that 11 genes/proteins are associated with biosynthesis of diaminopimelic acid (DAP), including 4-hydroxy-tetrahydrodipicolinate reductase (EC 1.17.1.8), 4-hydroxy-tetrahydrodipicolinate synthase (EC 4.3.3.7), aspartate-semialdehyde dehydrogenase (EC 1.2.1.11), aspartokinase (EC 2.7.2.4), diaminopimelate decarboxylase (EC 4.1.1.20), diaminopimelate epimerase (EC 5.1.1.7), L,L-diaminopimelate aminotransferase (EC 2.6.1.83), *N*-acetyl-L,L- diaminopimelate deacetylase (EC 3.5.1.47), *N*-succinyl-L,L- diaminopimelate desuccinylase (EC 3.5.1.18), UDP-*N*-acetylmuramoylalanyl-D-glutamate- 2,6-diaminopimelate ligase (EC 6.3.2.13), and UDP-*N*-acetylmuramoylalanyl-D-glutamyl-2,6-diaminopimelate-D-alanyl-D-alanine ligase (EC 6.3.2.10); 31 genes/proteins are associated with biosynthesis of polar lipids, including 1-acyl-*sn*-glycerol-3-phosphate acyltransferase (EC 2.3.1.51), acyl carrier protein (four copies), acyl-phosphate:glycerol-3-phosphate *O*-acyltransferase PlsY, alcohol dehydrogenase (EC 1.1.1.1) (eight copies), acetaldehyde dehydrogenase (EC 1.2.1.10) (two copies), aldehyde dehydrogenase (EC 1.2.1.3), aldehyde dehydrogenase B (EC 1.2.1.22), cardiolipin synthetase (EC 2.7.8.-), CDP-diacylglycerol-glycerol-3-phosphate 3-phosphatidyltransferase (EC 2.7.8.5), diacylglycerol kinase (EC 2.7.1.107), dihydroxyacetone kinase family protein, glycerate kinase (EC 2.7.1.31), glycerol kinase (EC 2.7.1.30) (two copies), glycerol-1-phosphate dehydrogenase [NAD(P)] (EC 1.1.1.261) (two copies), glycerol-3-phosphate dehydrogenase (EC 1.1.5.3), glycerol-3-phosphate dehydrogenase [NAD(P)^+^] (EC 1.1.1.94), phosphate:acyl-ACP acyltransferase PlsX, and phosphatidate cytidylyltransferase (EC 2.7.7.41); 14 genes/proteins are associated with biosynthesis of polyamines, including agmatine deiminase (EC 3.5.3.12), agmatine/putrescine antiporter, agmatine catabolism (two copies), arginine decarboxylase (EC 4.1.1.19)/lysine decarboxylase (EC 4.1.1.18), carbamate kinase (EC 2.7.2.2), carboxynorspermidine dehydrogenase, putative (EC 1.1.1.-) and putrescine carbamoyltransferase (EC 2.1.3.6), putrescine transport ATP-binding protein PotA (TC 3.A.1.11.1), *S*-adenosylmethionine decarboxylase proenzyme (EC 4.1.1.50), prokaryotic class 1A and spermidine putrescine ABC transporter permease component PotB (TC 3.A.1.11.1), spermidine putrescine ABC transporter permease component potC (TC_3.A.1.11.1) (two copies), spermidine synthase (EC 2.5.1.16) and transcriptional regulator, MerR family, near polyamine transporter; four genes/proteins are associated with biosynthesis of teichoic and lipoteichoic acids, including 2-C-methyl-D-erythritol 4-phosphate cytidylyltransferase (EC 2.7.7.60), teichoic acid export ATP-binding protein TagH (EC 3.6.3.40), teichoic acid translocation permease protein TagG, and undecaprenyl-phosphate *N*-acetylglucosaminyl 1-phosphate transferase (EC 2.7.8.-); and three genes/proteins are associated with biosynthesis of lipopolysaccharides, including lipopolysaccharide biosynthesis protein RffA (two copies), lipopolysaccharide cholinephosphotransferase LicD1 (EC 2.7.8.-), and HtrA protease/chaperone protein. There are no genes responsible for biosynthesis of respiratory lipoquinones or mycolic acids. The comparison of genes associated with biosynthetic pathways from RAST annotation between AF73-05CM02^T^ and *C. minuta* DSM 22607^T^ is listed in Table 3 and Supplementary Table S2. The number and kind of genes associated with diaminopimelic acid, polar lipids, polyamines, and teichoic and lipoteichoic acid biosynthesis make strain AF73-05CM02^T^ distinguishable from the reference species, *C. minuta* DSM 22607^T^. The analysis of CAZymes revealed that the genome of strain AF73-05CM02^T^ and *C. minuta* DSM 22607^T^ contained carbohydrate-binding modules (CBM5, CBM48), glycosyl transferase genes (GT13, GT2, GT28, GT35, GT4, GT47, GT5, and GT51) and glycoside hydrolase genes (GH13, GH28, GH3, GH4, GH6, GH77, GH78) in common. However, the presence/absence of GT30, GH23, GT39, GH26, and GH73 can distinguish strain AF73-05CM02^T^ from *C. minuta* DSM 22607^T^ (Supplementary Figure S3).

**TABLE 3:** Number of genes associated with biosynthetic pathways from whole genome sequences of strain AF73-05CM02^T^ and *C. minuta* DSM 22607^T^ identified by RAST.

Genes responsible for biosynthesis	AF73-05CM02^T^	*C. minuta* DSM 22607^T^
Diaminopimelic acid	10	11
Polar lipids	31	37
Polyamines	14	16
Teichoic and lipoteichoic acids	4	5
Lipopolysaccharides	3	3

*Numbers of genes identified for mycolic acids and quinones (benzoquinones and naphthoquinones) were zero for all taxa studied.*

**FIGURE 4:**
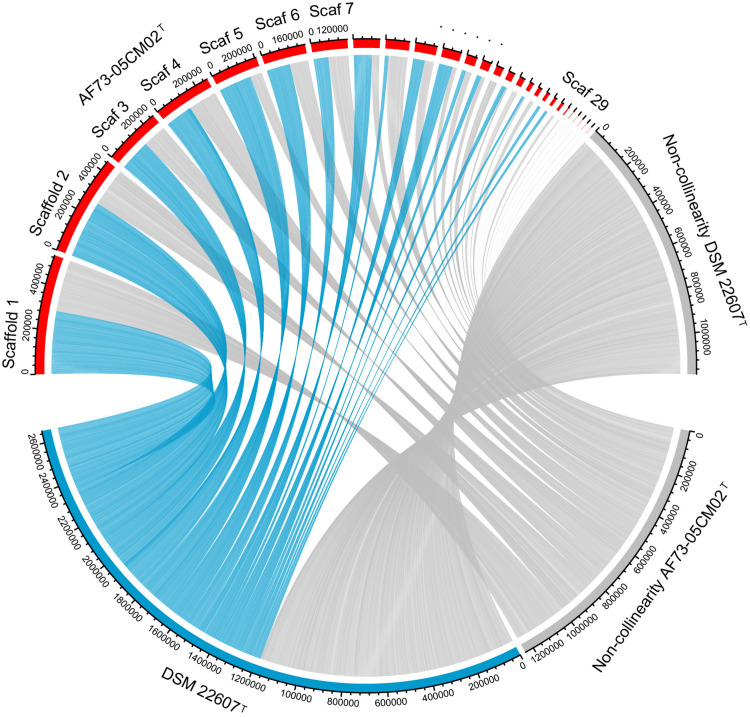
Genomic synteny shared between strains AF73-05CM02^T^ and *Christensenella minuta* DSM 22607^T^. The blue links represent synteny block and are internally independent from genomic rearrangement. The gray block represents the non-collinear region.

In order to further distinguish strain AF73-05CM02^T^ from the phylogenetically related species, the genome comparison was performed using BLAST average nucleotide identities (ANIb) and digital DNA–DNA hybridization (dDDH). The ANI and dDDH values between strain AF73-05CM02^T^ and the related reference species, *C. minuta* DSM 22607^T^, *C. hongkongensis* HKU16^T^, “*C. massiliensis*” Marseille-P2438, and “*C. timonensis*” Marseille-P2437 ranged from 78.76 to 83.51% and 20.20 to 26.80%, respectively (Table 1). The ANI and dDDH values comparing strain AF73-05CM02^T^ with the related species were significantly below the cutoff of 95–96 and 70%, respectively, which are proposed as threshold values for species the delineation in bacterial taxonomy (Goris et al., 2007), indicating that strain AF73-05CM02^T^ is a distinct species and should be classified as a representative of a novel species. The genome-wide collinearity analysis revealed a low degree of genome collinearity between strain AF73-05CM02^T^ and *C. minuta* DSM 22607^T^, with only 1,359 collinear genes and 33 collinear regions detected for each pair (Figure 4).

### Phenotypic Features

Strain AF73-05CM02^T^ is an obligate anaerobic and Gram-stain-negative bacterium. Cells are approximately 0.5 μm in width and 1.0–2.0 μm in length, occurring singly or in short chains. With phase contrast microscopy, cells were found to non-spore-forming, and flagella were not observed (Supplementary Figure S4). The bacteria formed punctiform colonies (approximately 0.2 mm in diameter) circular in form and beige in color after 4 days of growth at 37°C on PYG agar under anaerobic conditions. The growth temperature was from 30 to 42°C, with the optimum around 37–42°C, while no growth was observed below 30 or at 45°C. Growth occurred at pH values from 6.0 to 8.5, with optimum growth between pH 6.5 and 7.0. The strain tolerated salt concentrations up to 2% (w/v) NaCl and bile up to 0.3%. The cells were catalase negative. The physiological and biochemical comparisons of strain AF73-05CM02^T^ and the related strain were carried out using the API 20A, API 50CHL, and API ZYM tests, the results are summarized in the species description, and the differences of selected characteristics between our novel strain and the reference strain are given in Table 4. All the results of enzymatic characteristics and carbon source assimilation from the API ZYM, API 20A, and API 50CHL tests are presented in Supplementary Tables S3, S4.

**TABLE 4:** Comparison of phenotypic features between strain *C. intestinihominis* AF73-05CM02^T^ and the closest related reference strain, *C. minuta* DSM 22607^T^.

Phenotypic features	1*	2^#^
Cell size (μm)	1.0 × 1.0–2.0	0.4 × 0.8–1.9
**Growth:**		
Temperature range (optimum) (°C)	30–42 (37–42)	25–45 (37)
pH range (optimum)	6.0–8.5 (7.0)	6.0–9.0 (7.5)
Salt tolerance (%)	2	3
Bile tolerance (%)	0.3	20
Aesculin hydrolysis	+	–
**Acid from (API 20A and API 50CHL):**		
Arbutin	+	w
D-Galactose	+	w
D-Maltose	+	w (-^#^)
D-Sorbitol	+	w (-^#^)
D-Sucrose	+	–
D-Turanose	+	–
Gentiobiose	+	–
L-Sorbose	+	w
Xylitol	+	w
D-Adonitol	–	w
L-Fucose	–	+
D-Melezitose	W	–
D-Raffinose	W	–
**Enzyme activity (API ZYM):**		
β-Glucosidase	–	+

Strains: 1, C. intestinihominis sp. nov. AF73-05CM02^T^; 2, C. minuta DSM 22607^T^. +, Positive; w, weakly positive reaction; −, negative; ND, no data available. *Data from this study. ^#^Data from Morotomi et al. (2012) and this study.

Chemotaxonomic characteristics of strain AF73-05CM02^T^ were consistent with the results of the reference strain and were performed under identical conditions, confirming that the novel strain belongs to the genus *Christensenella*. The cellular fatty acid composition of strains AF73-05CM02^T^ and DSM 22607^T^ are presented in Table 5, and the dominant fatty acids (representing > 5% of the total) for strain AF73-05CM02^T^ are C_10__:__0_ (7.5%), iso-C_11__:__0_ (5.6%), C_12__:__0_ (7.2%), C_14__:__0_ (46.6%), iso-C_15__:__0_ (7.4%), C_16__:__0_ (9.7%), and C_18__:__1_ ω9*c* (6.9%). The higher amount of C_14__:__0_ and lower amounts of iso-C_15__:__0_ and C_16__:__0_ significantly differentiated strain AF73-05CM02^T^ from the reference strains. The cell wall diamino acid of strain AF73-05CM02^T^ is *meso*-diaminopimelic acid. The polar lipid profiles of strains AF73-05CM02^T^ and DSM 22607^T^ are shown in Supplementary Figure S5. The polar lipids of strain AF73-05CM02^T^ comprise diphosphatidylglycerol (DPG), phosphatidylglycerol (PG), three unidentified aminophospholipid (APL1–APL3), and three unidentified lipids (L1–L3). This polar lipid pattern is similar to the most closely related strain DSM 22607^T^, in which DPG, PG, and several unidentified lipids (L1 and L2) are present in both strains. However, the presence/absence of three unidentified aminophospholipid (APL1–APL3), two unidentified glycolipid (GL1 and GL2), phospholipid (PL1 and PL2), and an unidentified lipid (L3) can be used to distinguish strain AF73-05CM02^T^ from its closest relative. Quinones were not detected.

**TABLE 5:** Cellular fatty acid composition of strain AF73-05CM02^T^ and a closely related species, DSM 22607^T^.

Fatty acids	1	2
C_10:0_	**7.5**	**8.6**
C_12:0_	**7.2**	1.1
C_14:0_	**46.6**	**13.0**
C_14:0_ 2OH	t	1.3
C_16:0_	**9.7**	**21.1**
C_18:1_ ω9*c*	**6.9**	**6.8**
C_18:1_ ω7*c*	t	3.9
C_18:0_	1.8	3.7
Iso-C_11:0_	**5.6**	2.9
Iso-C_15:0_	**7.4**	**27.4**
Anteiso-C_11:0_	t	1.3
Anteiso-C_13:0_	t	2.4
Anteiso-C_15:0_	1.3	3.2
Iso-C_17:1_ I/anteiso B	4.7	1.7
Antei-C_18:0_/C_18:2_ ω6,9*c*	t	1.7

*Strains: 1, AF73-05CM02^T^; 2, C. minuta DSM 22607^T^; data were obtained in this study. Numbers represent percentages of the total fatty acids. Only fatty acids amounting to 1% or higher are shown. t, traces (<1%).*

Metabolic end products from glucose for strains AF73-05CM02^T^ and DSM 22607^T^ are shown in Supplementary Table 5. Acetic acid, formic acid, butyric acid, and lactic acid were the major end products (>1 mmol/L) for strain AF73-05CM02^T^.

We found that strain AF73-05CM02^T^ can be clearly differentiated from *C. minuta* DSM 22607^T^ based on several phenotypic and genotypic characteristics and ANI values, which suggest that strain AF73-05CM02^T^ represents a novel species of the genus *Christensenella.* Therefore, we propose AF73-05CM02^T^ (=CGMCC 1.5207^T^ = DSM 103477^T^ ) as the type strain of *Christensenella intestinihominis* sp. nov.

### Safety Evaluation

Safety evaluation is an essential step of new candidate probiotic for human and animal applications. In our study, we examined the antibiotic susceptibility and hemolytic activity *in vitro* and analyzed virulence factor genes based on the genome sequence of strain AF73-05CM02^T^. In antibiotic susceptibility tests, strain AF73-05CM02^T^ was resistant to oxacillin and sulfamethoxazole, but sensitive to penicillin, ampicillin, carbenicillin, piperacillin, vancomycin, polymyxin B, furazolidone, chloramphenicol, and clindamycin (Supplementary Table S6). The hemolytic activity of the cells was not detected as indicated by a lack of clear zone formation. Strain AF73-05CM02^T^ harbors no virulence factor genes.

### Beneficial Potential for EPS Production and Cholesterol Reduction

EPS produced by probiotic bacteria has several biologically beneficial functions on the host, such as improving the viscosity of the lactic acid bacteria-fermented products (Li et al., 2014), and has significant roles in colonization, stress resistance, and adhesion (Delcour et al., 1999). Furthermore, it has been suggested that EPS may have probiotic properties in relation to immune modulation and antioxidative effects (Welman and Maddox, 2003; Fanning et al., 2012). In the present research, both test strains *C. intestinihominis* AF73-05CM02^T^ and *C. minuta* DSM 22607^T^ were capable of producing EPS in amounts of 234 and 271 mg/L, respectively. To better understand the biosynthetic pathway involved in EPS production in strains AF73-05CM02^T^ and *C. minuta* DSM 22607^T^, we analyzed the CAZymes associated with synthesis of EPS. Interestingly, enzymes belonging to CBM5, GH3, GT2, and GT4 were present in the genomes of both strains AF73-05CM02^T^ and *C. minuta* DSM 22607^T^, whereas GH26 and GT39 were present only in the genome of *C. minuta* DSM 22607^T^.

The cholesterol-lowering activity was determined in PYG-CHO broth supplemented with bile. Both strains AF73-05CM02^T^ and *C. minuta* DSM 22607^T^ showed a capacity for eliminating cholesterol from the PYG-CHO broth. After incubation in PYG-CHO at 37°C for 4 days, the amounts of cholesterol in the medium were reduced with efficiency of 36.6 and 54.3% by strains AF73-05CM02^T^ and *C. minuta* DSM 22607^T^, respectively. The control sample, containing no cultures, demonstrated as expected no change in cholesterol content. Several hypotheses have been proposed to explain the cholesterol-lowering ability of probiotics, including deconjugated bile acids via bile salt hydrolase activity, adsorption to cellular surface, and conversion by probiotics (Ishimwe et al., 2015). To further investigate the potential mechanisms behind a cholesterol-lowering ability, we explored genes related to cholesterol metabolism in the genome of strains AF73-05CM02^T^ and *C. minuta* DSM 22607^T^. From the annotation data from KEGG, six KO, namely, K16045, K14674, K12298, K03333, K01052, and K00637, related to cholesterol metabolism were present in the genomes of both strains AF73-05CM02^T^ and *C. minuta* DSM 22607^T^. Strain *C. minuta* DSM 22607^T^ contained one more KO, K00637, compared to strain AF73-05CM02^T^ (Supplementary Table S7).

In a previous study, *in vivo* experiments exploiting the cholesterol-lowering effect showed that the probiotics was effective and safe for modulating the serum-lipid profile and reducing the host cholesterol level (Pan et al., 2010). A high cholesterol level as a consequence of obesity can increase the risk of CVDs. The genus *Christensenella* has been found in high abundance in the gut of lean individuals (Goodrich et al., 2014), suggesting that *Christensenella* has a potential for protecting against obesity. Further studies will be required to elucidate the cholesterol-reducing properties *in vitro* and *in vivo* and the potential for using *Christensenella* as a probiotics.

### Description of *C. intestinihominis* sp. nov.

*C. intestinihominis* (in.tes.ti.ni.ho’mi.nis. L. gen. n. *intestini* of the intestine; L. gen. n. *hominis* of a human being; N.L. gen. n. *intestinihominis* of the human intestine).

Cells are Gram-stain-negative, obligate anaerobic, non-motile, short rods (1.0 × 1.0–2.0 μm) isolated from a fecal sample collected from a healthy adult. Colonies on PYG agar are 0.2 mm in diameter and punctiform with a circular shape and beige color after 4 days of growth at 37°C. Growth occurs at temperatures from 30 to 42°C, with an optimum around 37–42°C. The pH range is from 6.0 to 8.5, with an optimum between pH 6.5 and 7.0. Colonies are able to grow in the presence of up to 2.0% (w/v) NaCl and 0.3% bile (w/v). Major end products of metabolism of glucose are acetic acid, formic acid, butyric acid, and lactic acid. The cells exhibit resistance to oxacillin and sulfamethoxazole but are sensitive to penicillin, ampicillin, carbenicillin, piperacillin, vancomycin, polymyxin B, furazolidone, chloramphenicol, and clindamycin. The predominant cellular fatty acids are C_10__:__0_, iso-C_11__:__0_, C_12__:__0_, C_14__:__0_, iso-C_15__:__0_, C_16__:__0_, and C_18__:__1_ ω9*c*. The diagnostic cell wall diamino acid is LL-diaminopimelic acid.

In API 20A and API 50CHL tests, the strain was positive for utilization of arbutin, D-arabinose, D-fructose, D-fucose, D-galactose, D-glucose, D-lyxose, D-ribose, D-sorbitol, D-sucrose, D-tagatose, D-turanose, D-xylose, gentiobiose, L-arabinose, L-rhamnose, L-sorbose, methyl-β-D-xylopyranoside, salicin, and xylitol, has weak reactions for D-maltose, D-mannose, D-melezitose, D-raffinose, erythritol, L-xylose, and salicin, and was negative for amygdalin, cellobiose, D-adonitol, D-arabitol, D-lactose, D-mannitol, D-melibiose, D-trehalose, dulcitol, gluconate, glycerol, glycogen, inositol, inulin, L-arabitol, L-fucose, methyl-D-glucopyranoside, methyl-α-D-mannopyranoside, *N*-acetyl-glucosamine, 2-ketogluconate, and 5-ketogluconate. Indole is not formed. Esculin can be degraded, but gelatin is not hydrolyzed. Catalase is negative. Results obtained from API ZYM showed positive enzymatic activity on naphthol-AS-BI-phosphohydrolase and negative results for alkaline phosphatase, esterase (C4), esterase lipase (C8), lipase (C14), leucine arylamidase, valine arylamidase, cystine arylamidase, trypsin, α-chymotrypsin, acid phosphatase, α-galactosidase, β-galactosidase, β-glucuronidase, α-glucosidase, β-glucosidase, *N*-acetyl-β-glucosaminidase, α-mannosidase, and β-fucosidase. The polar lipids comprise DPG, PG, three APLs, and three Ls.

The type strain AF73-05CM02^T^ (=CGMCC 1.5207^T^ = DSM 103477^T^ ) was isolated from the fecal samples of a healthy adult residing in Shenzhen, China. The DNA G + C content of strain AF73-05CM02^T^ is 52.07 mol% calculated from the genome sequence. The genome size is 3.02 Mbp.

## Data Availability Statement

The GenBank/EMBL/DDBJ accession number for the 16S rRNA gene sequence of *Christensenella intestinihominis* AF73-05CM02^T^ is KX078376. The draft genome of *C. intestinihominis* AF73-05CM02^T^ has been deposited at DDBJ/EMBL/GenBank under the accession number MAIQ00000000. The data that support the findings of this study have also been deposited into CNGB Sequence Archive (CNSA) (Guo et al., 2020) of China National GeneBank DataBase (CNGBdb) (Chen et al., 2020) with accession number CNPhis0003415.

## Ethics Statement

The studies involving human participants were reviewed and approved by Institutional Review Board on Bioethics and Biosafety of BGI. The patients/participants provided their written informed consent to participate in this study.

## Author Contributions

YZ and LX conceived and designed the experiments. YZ, WX, ML, S-WL, and YD performed the experiments. YZ, LX, GL, TH, C-HS, and XL analyzed the data. YZ, WX, ML, and YD contributed reagents, materials, and analysis tools. YZ wrote the manuscript. KK revised the manuscript.

## Conflict of Interest

The authors declare that the research was conducted in the absence of any commercial or financial relationships that could be construed as a potential conflict of interest.

## Footnotes

1 https://www.ezbiocloud.net/

2 http://ggdc.dsmz.de/distcalc2.php

3 http://stothard.afns.ualberta.ca/cgview_server/index.html

## Supplementary Material

Supplementary Figure S1 Neighbor-joining phylogenetic tree based on 16S rRNA gene sequences showing the phylogenetic relationships of strains AF73-05CM02^T^ and the representatives of related taxa. *Bacillus subtilis subsp. subtilis* NCIB 3610^T^ (ABQL01000001) was used as an out-group. Bootstrap values based on 1,000 replications higher than 70% are shown at the branching points. Bar, substitutions per nucleotide position.

Supplementary Figure S2 Minimum-evolution phylogenetic tree based on 16S rRNA gene sequences showing the phylogenetic relationships of strains AF73-05CM02^T^ and the representatives of related taxa. *Bacillus subtilis* subsp. *subtilis* NCIB 3610^T^ (ABQL01000001) was used as an out-group. Bootstrap values based on 1,000 replications higher than 70% are shown at the branching points. Bar, substitutions per nucleotide position.

Supplementary Figure S3 Venn diagram of the CAZymes for the comparison of strain AF73-05CM02^T^ and the reference strain *C. minuta* DSM 22607^T^.

Supplementary Figure S4 Gram staining of strain AF73-05CM02^T^.

Supplementary Figure S5 Two-dimensional TLC separation of polar lipids of strain AF73-05CM02^T^ and the reference strain *C. minuta* DSM 22607^T^. Total polar lipids were stained with molybdatophosphoric acid. DPG, diphosphatidylglycerol; PG, phosphatidylglycerol; PL, unidentified phospholipid; L, unidentified lipid; APL, unidentified aminophospholipid; L, unidentified lipids; GL, unidentified glycolipid.

Supplementary Figure S6 Certification. Deposit certification of CGMCC.

Supplementary Figure S7 Certification. Deposit certification of DSMZ.

Supplementary Table S1 Number of genes associated with general COG functional categories in the genome of *C. intestinihominis* AF73-05CM02^T^ and *C. minuta* DSM 22607^T^.

Supplementary Table S2 The specific genes/protein related to biosynthesis of DAP, polar lipids, polyamines and lipoteichoic and teichoic acids and their positions in the genome of strain AF73-05CM02^T^ identified by Rapid Annotation Subsystem Technology (RAST).

Supplementary Table S3 Enzymatic characteristics of strain AF73-05CM02^T^ based on API ZYM tests.

Supplementary Table S4 Carbon source assimilation of strain AF73-05CM02^T^ from API 20A and API 50CHL test.

Supplementary Table S5 Antibiotic sensitivity of strain AF73-05CM02^T^.

Supplementary Table S6 Metabolic end products from glucose for strain AF73-05CM02^T^ and *C. minuta* DSM 22607^T^.

Supplementary Table S7 List of KO related to cholesterol metabolism in the genome of strain AF73-05CM02^T^ and *C. minuta* DSM 22607^T^.
