# Supplementary figures and images for: Taxonomic Description and Genome Sequence of Christensenella intestinihominis sp. nov., a Novel Cholesterol-Lowering Bacterium Isolated From Human Gut
Source: Front Microbiol. 2021 Feb 22;12:632361. doi: 10.3389/fmicb.2021.632361 (PMC7937921; doi:10.3389/fmicb.2021.632361)

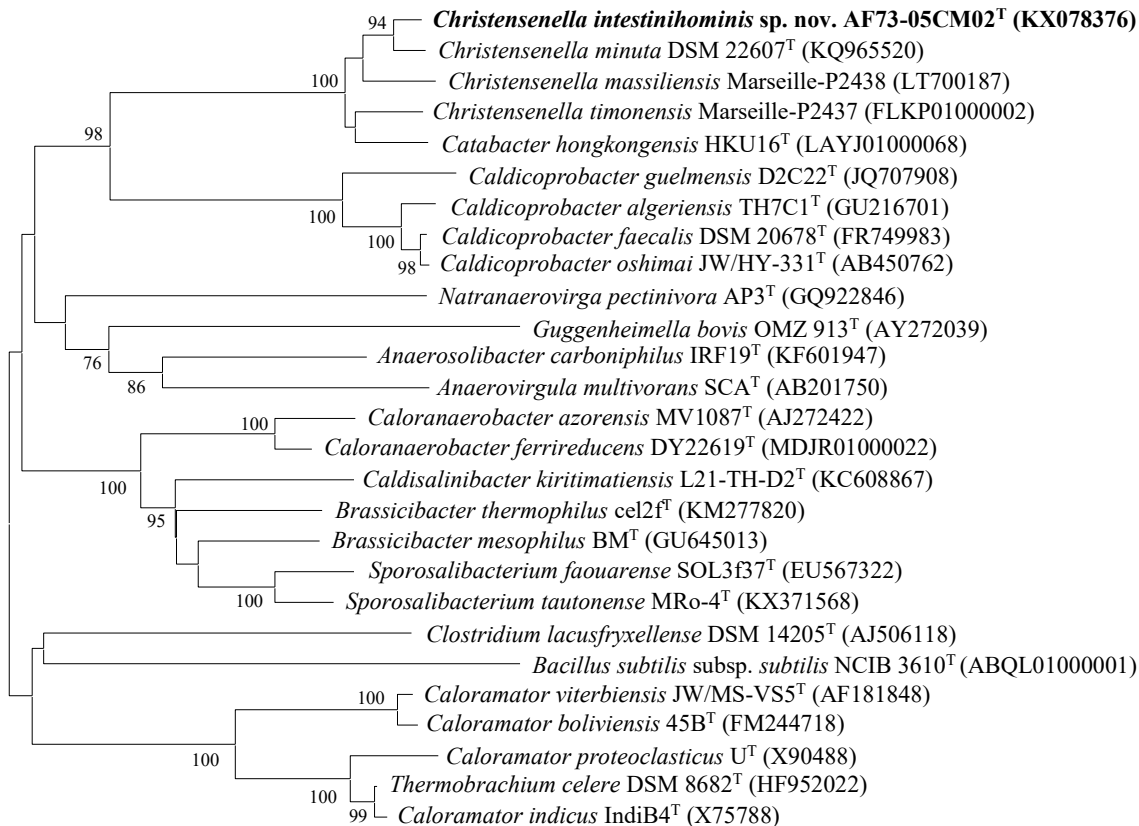

0.020

Supplementary Figure S1

Supplement: Supplementary Figure S1 — Neighbor-joining phylogenetic tree based on 16S rRNA gene sequences showing the phylogenetic relationships of strains AF73-05CM02T and the representatives of related taxa. Bacillus subtilis subsp. subtilis NCIB 3610T (ABQL01000001) was used as an out-group. Bootstrap values based on 1,000 replications higher than 70% are shown at the branching points. Bar, substitutions per nucleotide position. [file Data_Sheet_1.PDF]

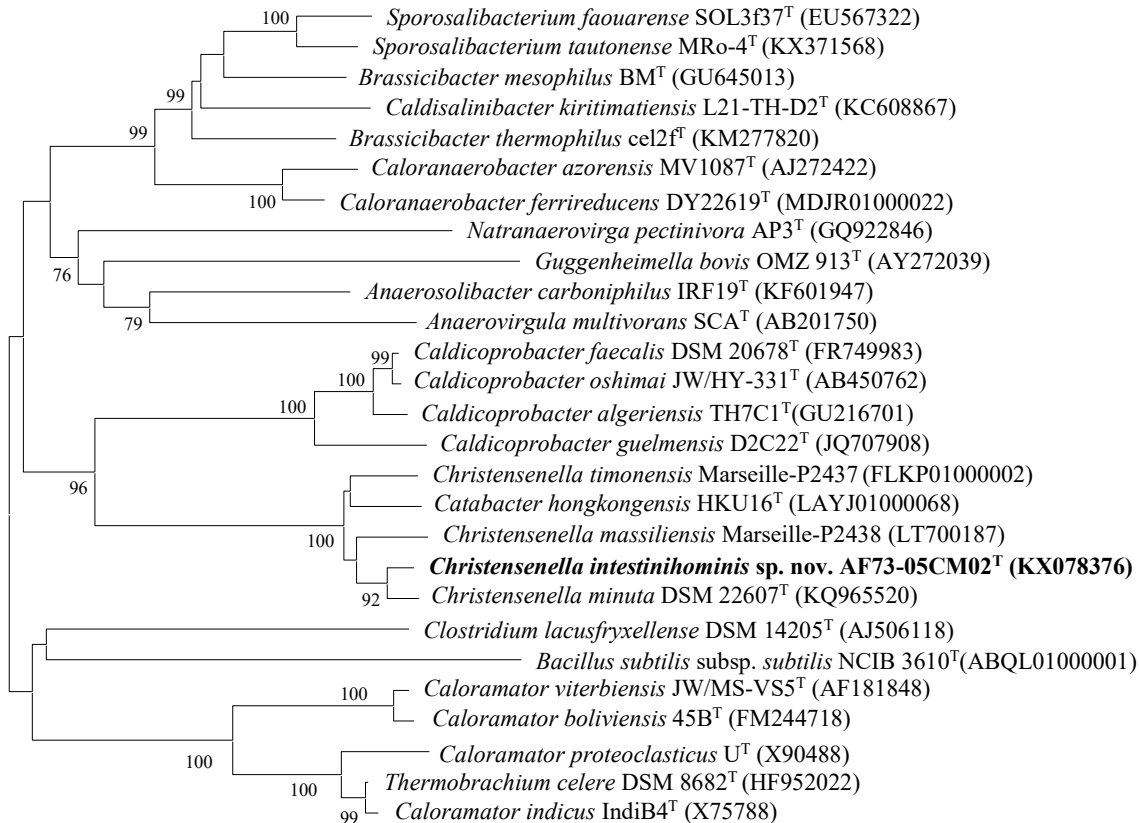

0.020

Supplementary Figure S2

Supplement: Supplementary Figure S2 — Minimum-evolution phylogenetic tree based on 16S rRNA gene sequences showing the phylogenetic relationships of strains AF73-05CM02T and the representatives of related taxa. Bacillus subtilis subsp. subtilis NCIB 3610T (ABQL01000001) was used as an out-group. Bootstrap values based on 1,000 replications higher than 70% are shown at the branching points. Bar, substitutions per nucleotide position. [file Data_Sheet_2.PDF]

AF73-05CM02<sup>T</sup>

DSM 22607<sup>T</sup>

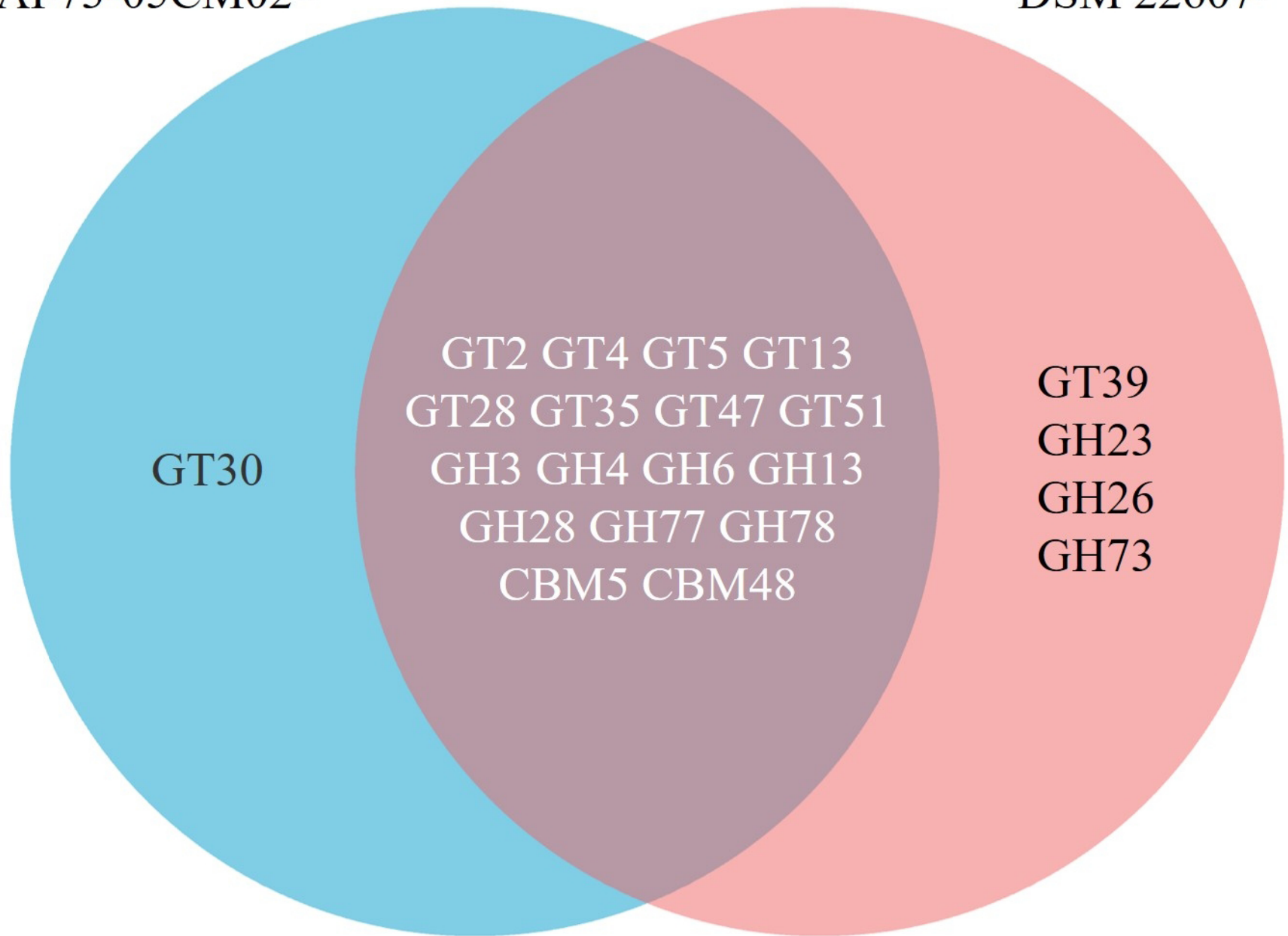

Supplement: Supplementary Figure S3 — Venn diagram of the CAZymes for the comparison of strain AF73-05CM02T and the reference strain C. minuta DSM 22607T. [file Data_Sheet_3.PDF]

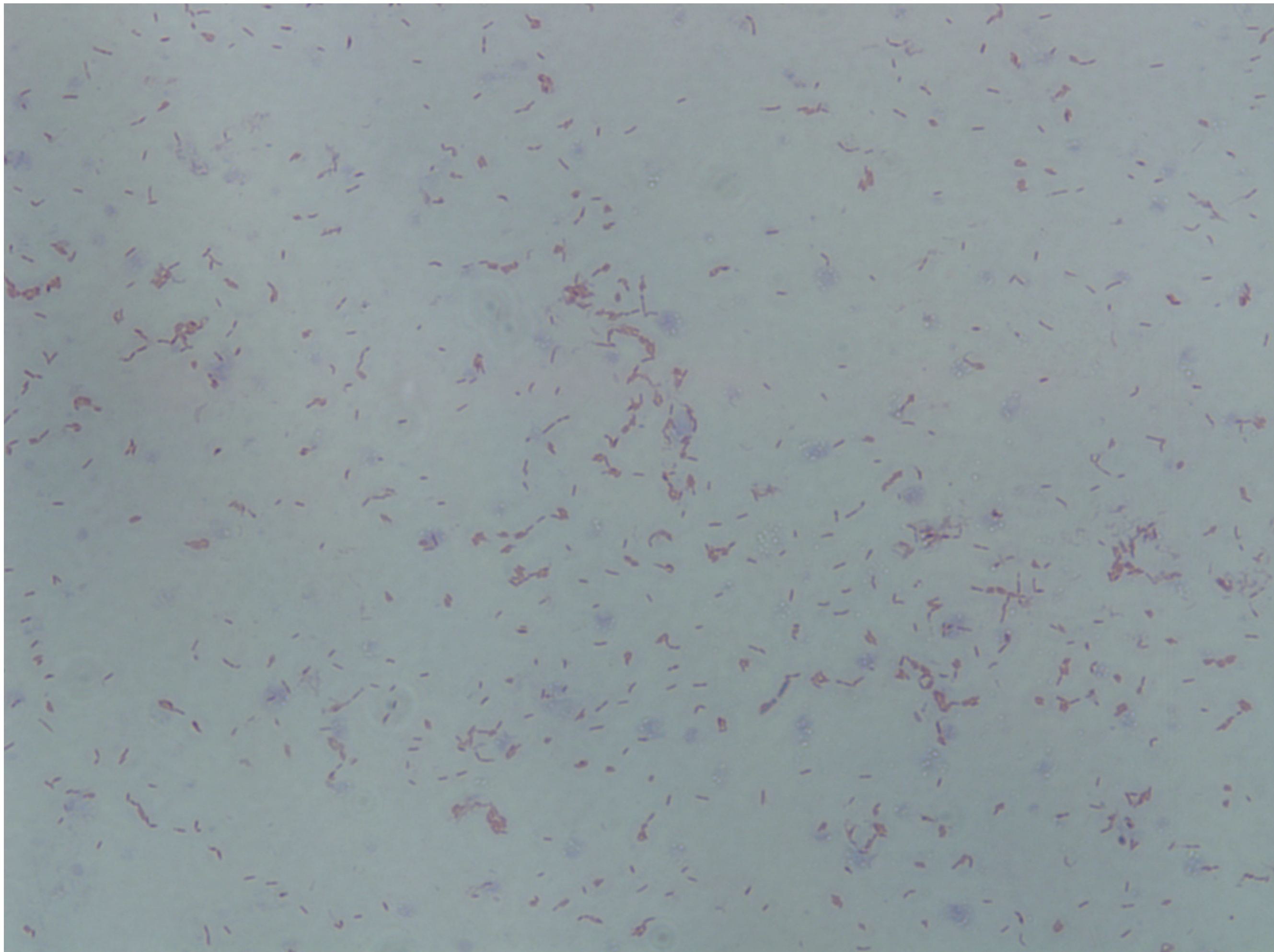

Supplementary Figure S4

Supplement: Supplementary Figure S4 — Gram staining of strain AF73-05CM02T. [file Data_Sheet_4.PDF]

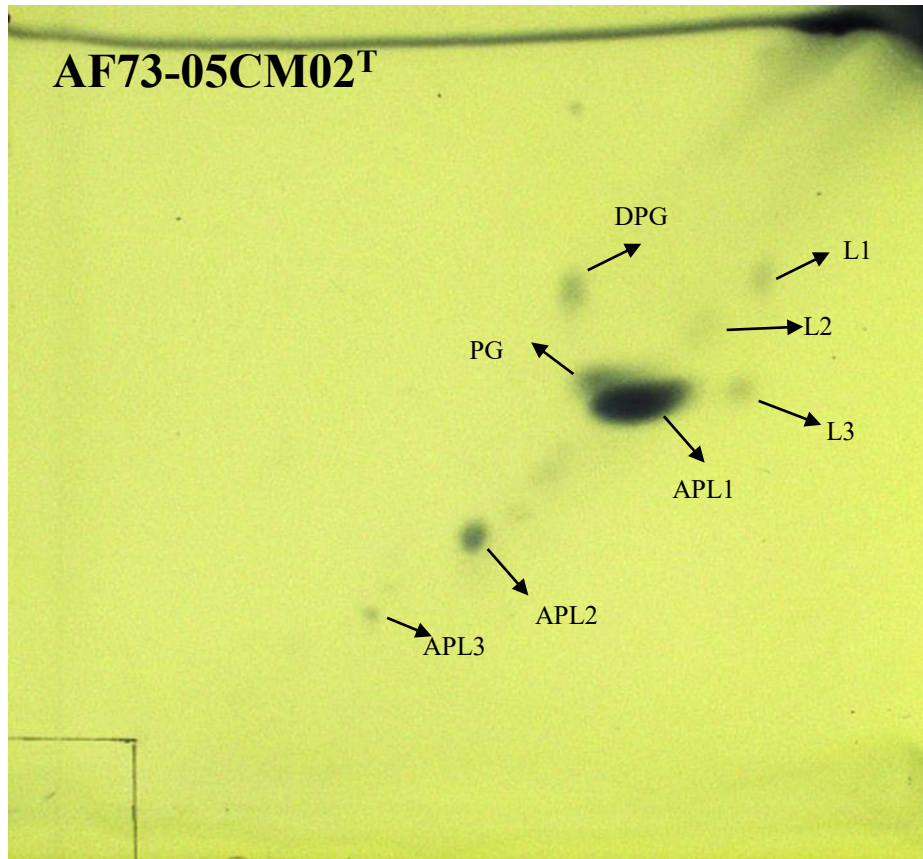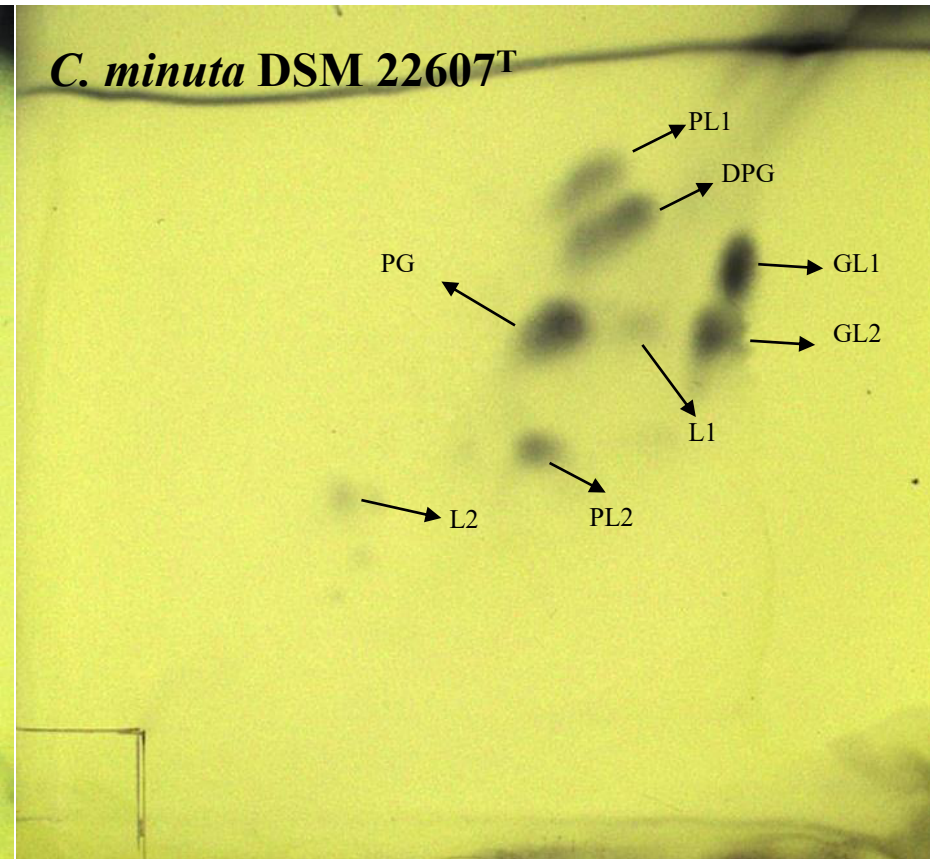

**Supplementary Figure S5**

Supplement: Supplementary Figure S5 — Two-dimensional TLC separation of polar lipids of strain AF73-05CM02T and the reference strain C. minuta DSM 22607T. Total polar lipids were stained with molybdatophosphoric acid. DPG, diphosphatidylglycerol; PG, phosphatidylglycerol; PL, unidentified phospholipid; L, unidentified lipid; APL, unidentified aminophospholipid; L, unidentified lipids; GL, unidentified glycolipid. [file Data_Sheet_5.PDF]
