# Supplementary material for: Taxonomic Description and Genome Sequence of Christensenella intestinihominis sp. nov., a Novel Cholesterol-Lowering Bacterium Isolated From Human Gut
Source: Front Microbiol. 2021 Feb 22;12:632361. doi: 10.3389/fmicb.2021.632361 (PMC7937921; doi:10.3389/fmicb.2021.632361)
Supplement: Supplementary Figure S6 — Certification. Deposit certification of CGMCC. [file Data_Sheet_6.PDF]

中国微生物菌种保藏管理委员会普通微生物中心
China General Microbiological Culture Collection Center (CGMCC)

Address: Institute of Microbiology, Chinese Academy of Sciences, Datun Road, Chaoyang District, Beijing 100101, China
Telephone: 86-10-64807355 Fax: 86-10-64807288 E-mail: cgmcc@im.ac.cn Homepage: www.cgmcc.net

受理通知书

NOTIFICATION OF RECEIPT

CGMCC 1.5207

1. Name and address of the depositor or agent

   薛文斌 Wen-Bin XUE
   BGI-Shenzhen
   Main Building, Beishan Industrial Zone, Yantian District, Shenzhen 518083, China

2. Strain reference given by depositor

   AF73-05CM02

3. Deposited microorganisms appended

   ☐ Scientific description

   ■ Proposed taxonomic name

   Christensenella intestinihominis

4. The deposited microorganism has been received and numbered as CGMCC 1.5207 on January, 2016. The strain has been checked for viability in the CGMCC and is stored using one of the standard methods used in the CGMCC.

5. This strain is available in the public accessible section of the CGMCC and restrictions have not been placed on access. It will be included in the published and online catalogue after publication of this number by the authors.

Signature of Head of CGMCC Yu-Guang ZHOU

Date June 13, 2016

中国微生物菌种保藏管理委员会 普通微生物中心
