# Supplementary material for: Taxonomic Description and Genome Sequence of Christensenella intestinihominis sp. nov., a Novel Cholesterol-Lowering Bacterium Isolated From Human Gut
Source: Front Microbiol. 2021 Feb 22;12:632361. doi: 10.3389/fmicb.2021.632361 (PMC7937921; doi:10.3389/fmicb.2021.632361)
Supplement: Supplementary Figure S7 — Certification. Deposit certification of DSMZ. [file Data_Sheet_7.PDF]

Braunschweig
Sep. 20, 2016

Confirmation of the availability of a strain for the purpose of valid publication of a new name according to the Bacteriological Code

The following information is confidential and serves only to allow the International Journal of Systematic and Evolutionary Microbiology to confirm that a strain has been deposited and will be available from the DSMZ in accordance with the Rules of the Bacteriological Code (1990 revision) as revised by the ICSP at the plenary sessions in Sydney and Paris.

Strain AF73-05CM02 has been deposited in the DSMZ under the number

DSM 103477

This strain is available in the publically accessible section of the DSMZ and restrictions have not been placed on access to information concerning the presence of this strain in the DSMZ. It will be included in published and online catalogues after publication of this number by the authors.

This strain has been checked for viability in the DSMZ and is stored using one of the standard methods used in the DSMZ.

The depositor of this strain has also carried out a "depositor's check" and confirmed the identity of the strain held under this DSM number.

The DSMZ is not responsible for differences between the properties of the strain deposited in the DSMZ and properties given in the literature/databases.
It is the sole responsibility of the depositor to ensure that type strains deposited in the DSMZ conform to the requirements of the appropriate Rules governing prokaryotes nomenclature and the deposition of type strains (Rules 18a, 27, & 30 of the ICNB/ICNP, including changes made at plenary sessions of the JC/ICSP).

Stefan Spring

Dr. Stefan Spring
Curator responsible for the strain
