# Supplementary material for: Taxonomic Description and Genome Sequence of Christensenella intestinihominis sp. nov., a Novel Cholesterol-Lowering Bacterium Isolated From Human Gut
Source: Front Microbiol. 2021 Feb 22;12:632361. doi: 10.3389/fmicb.2021.632361 (PMC7937921; doi:10.3389/fmicb.2021.632361)
Supplement: Supplementary Table S5 — Antibiotic sensitivity of strain AF73-05CM02T. [file Table_5.docx]

**Supplementary Table S5. Antibiotic sensitivity of strain AF73-05CM02^T^.**

| **NO.** | **Antibiotic** | **Concentration (μg)** | **AF73-05CM02^T^** |
| --- | --- | --- | --- |
| 1 | Penicillin | 10 | S |
| 2 | Oxacillin | 1 | R |
| 3 | Ampicillin | 10 | S |
| 4 | Carbenicillin | 100 | S |
| 5 | Piperacillin | 100 | S |
| 6 | Vancomycin | 30 | S |
| 7 | Polymyxin B | 300 IU | S |
| 8 | Compound sulfamethoxazole | 25 | R |
| 9 | Furazolidone | 300 | S |
| 10 | Chloroamphenicol | 30 | S |
| 11 | Clindamycin | 2 | S |

R, resistant; S, sensitive.
